# Silver nanoparticles inhibit VEGF-and IL-1β-induced vascular permeability via Src dependent pathway in porcine retinal endothelial cells

**DOI:** 10.1186/1477-3155-7-8

**Published:** 2009-10-30

**Authors:** Sardarpasha Sheikpranbabu, Kalimuthu Kalishwaralal, Deepak Venkataraman, Soo Hyun Eom, Jongsun Park, Sangiliyandi Gurunathan

**Affiliations:** 1 Department of Biotechnology, Division of Molecular and Cellular Biology, Kalasalingam University (Kalasalingam Academy of Research and Education), Anand Nagar, Krishnankoil-626190, Tamilnadu, India; 2 Department of Life Science, Cell Dynamics Research Center & Systems Biology Research Center, Gwangju Institute of Science and Technology, Gwangju 500-712, South Korea; 3 Cell Signaling Laboratory, Cancer Research Institute, Department of Pharmacology, College of Medicine, Chungnam National University, 6 Munhwa-dong, Jung-gu, Taejon, 301-131, South Korea

## Abstract

The aim of this study is to determine the effects of silver nanoparticles (Ag-NP) on vascular endothelial growth factor (VEGF)-and interleukin-1 beta (IL-1β)-induced vascular permeability, and to detect the underlying signaling mechanisms involved in endothelial cells. Porcine retinal endothelial cells (PRECs) were exposed to VEGF, IL-1β and Ag-NP at different combinations and endothelial cell permeability was analyzed by measuring the flux of RITC-dextran across the PRECs monolayer. We found that VEGF and IL-1β increase flux of dextran across a PRECs monolayer, and Ag-NP block solute flux induced by both VEGF and IL-1β. To explore the signalling pathway involved VEGF- and IL-1β-induced endothelial alteration, PRECs were treated with Src inhibitor PP2 prior to VEGF and IL-1β treatment, and the effects were recorded. Further, to clarify the possible involvement of the Src pathways in endothelial cell permeability, plasmid encoding dominant negative(DN) and constitutively active(CA) form of Src kinases were transfected into PRECs, 24 h prior to VEGF and IL-1β exposure and the effects were recorded. Overexpression of DN Src blocked both VEGF-and IL-1β-induced permeability, while overexpression of CA Src rescues the inhibitory action of Ag-NP in the presence or absence of VEGF and IL-1β. Further, an *in vitro *kinase assay was performed to identify the presence of the Src phosphorylation at Y419. We report that VEGF and IL-1β-stimulate endothelial permeability via Src dependent pathway by increasing the Src phosphorylation and Ag-NP block the VEGF-and IL-1β-induced Src phosphorylation at Y419. These results demonstrate that Ag-NP may inhibit the VEGF-and IL-1β-induced permeability through inactivation of Src kinase pathway and this pathway may represent a potential therapeutic target to inhibit the ocular diseases such as diabetic retinopathy.

## Background

Vascular endothelial barrier dysfunction occurs in a large number of disease processes including diabetic retinopathy, stroke, pulmonary edema, myocardial infarction, inflammatory bowel disease, nephropathies, rheumatoid arthritis, and tumours. In these diseases, increased vascular permeability is associated with elevated levels of either one or more growth factors or cytokines [1]. Vascular endothelial growth factor (VEGF) has received considerable attention as a tumour-secreted vascular permeability factor [2,3]. VEGF is determined to posses 50,000 times more potency than histamine in inducing vasopermeability in the dermal vasculature. Previous reports indicate the correlation between the increases in permeability in ischemic retinopathies and possibly also in exudative macular degeneration and uveitis and the increased VEGF levels [4-7]. In fact, VEGF antagonists have been successfully used to reduce retinal/macular edema in neovascular eye diseases such as age-related macular degeneration with stabilization or even improvement of visual acuity in a subset of affected patients [8]. Although VEGF is thought to play a major role in stimulating vascular permeability, this process undoubtedly involves multiple other factors as well, including inflammatory cytokines such as interleukin-1beta (IL-1β) [9]. Previously IL-1β was shown to induce the permeability through the vasculature of the blood retinal barrier in rats [10].

Now a great deal of research is focused on the development of inhibitors for vascular permeability. In fields like drug delivery, imaging and diagnosis & treatment of cancer various nanoparticles are proposed to function as a tool [11,12]. Furthermore, currently efforts are being made to investigate the use of nanomaterials in various therapeutic applications, where the nanoparticles could be the active component or could just be the physical support for the functional moieties. In addition, the importance of augmenting the performance of conventional drugs by incorporating the nanoparticles cannot be overstated as the synergistic effect may offer valuable alternatives with minimization of harmful consequences. Therefore, the development of novel therapeutic strategies that specifically target diabetic retinopathy is desired for patients with diabetes. As the size of the smallest capillary is in the order of 5-6 μm, nanomaterials are highly advantageous in this regard as their size allows exceptional access to targets at various parts of human body. Studies have shown that the properties of the nanoparticles vary according to the cell types. Ultrafine particles (1-10 nm) are found to cause inflammatory responses, where as relatively larger particles (50 nm) are internalized readily through the endothelial cells without much toxicity [13-15]. A recent study reported that intravesical administration of nanocrystalline silver (1%) has decreased the levels of urine histamine, bladder tumour necrosis factor-alpha and mast cell activation without any toxic effect. This action might be useful for interstitial cystitis [16]. In addition, it has been suggested that the effect of NPI 32101 on suppression of inflammatory cytokines and MMP-9 may be responsible for its anti-inflammatory activity [17].

Endothelial cells play a central role in angiogenesis, carcinogenesis, atherosclerosis, myocardial infarction, limb and cardiac ischemia, and tumour growth [18,19]. Endothelium is an important target for various drug and gene therapy. The vascular endothelial monolayer forms a semi-selective permeability barrier between blood and the interstitial space to control the movement of blood fluid, proteins, and macromolecules across the vessel wall. Alteration of permeability barrier integrity plays a major role in drug-based therapies, as well as the pathogenesis of cardiovascular diseases, inflammation, acute lung injury syndromes, and carcinogenesis [20,21].

Solute flux assay has been successfully employed to study the effects of VEGF [22] and corticosteroids on retinal endothelial cell permeability. In the present study, we have investigated the molecular mechanism of silver nanoparticles on VEGF-and IL-1β- induced retinal endothelial cell permeability. We show that both VEGF and IL-1β increase endothelial cell permeability via Src dependent pathway. Silver nanoparticles were found to block VEGF-and IL-1β-induced permeability in retinal endothelial cells from porcine retina and this inhibitory effect was dependent on the modulation via Src phosphorylation at Y419. The results obtained in this study may provide some insights into the translocation pathways of nanoparticles in general.

## Materials and methods

### Biosynthesis of silver nanoparticles

In a typical experiment, 2 g of wet *Bacillus licheniformis *biomass was taken in an erlenmeyer's flask. 1 mM AgNO_3 _solution was prepared using deionized water and 100 ml of the solution mixture was added to the biomass. Then the conical flask was kept in a shaker at 37°C (200 rpm) for 24 h for the synthesis of nanoparticles [23,24].

### Characterization of silver nanoparticles

Silver nanoparticles were synthesized using *B. licheniformis*. The synthesized nanoparticles were primarily characterized by UV-Visible spectroscopy followed by XRD and Transmission electron microscopic analysis. Finally, the size distribution of the nanoparticles was evaluated using DLS measurements, which were conducted with a Malvern Zetasizer ZS compact scattering spectrometer (Malvern Instruments Ltd., Malvern, UK).

### Purification of nanoparticles

Bacteria were grown in a 1000 ml Erlenmeyer flask that contained 200 ml of nitrate medium. The flasks were incubated for 24 h in an environmental shaker set at 120 rpm and 37°C. After the incubation period, the culture was centrifuged at 4,000 × g and the supernatant used for the synthesis of silver nanoparticles. 1 mM of AgNO_3 _was mixed with 200 ml of cell filtrate in a 1000 ml Erlenmeyer flask. Bio-reduction was monitored by recording the UV-Vis absorption spectra as a function of time of the reaction mixture. The particles were washed five times by centrifugation and re-dispersed in water to remove excess of silver. They were then transferred to a dialysis tube with a 12,000 molecular weight cut off. Nanoparticles were resuspended in 1 ml of HEPES buffer (20 mM, pH 7.4) supplemented with sucrose to reach a density of 2.5 g/ml and gradient was made according to method described earlier [25-27]. The solution was placed at the bottom of a centrifuge tube (13 ml). Twelve millilitres of a linear gradient of sucrose (0.25-1 M) density was layered on the nanoparticle suspension and submitted to ultracentrifugation (200,000 *g *at 4°C for 16 h) by using an SW41 rotor (Beckman Instruments, Fullerton, CA, USA). Fractions (1 ml) were collected and purified sample was further characterized by UV-Vis and TEM. The purified Ag-NP was utilized for further experiments.

### Cell culture

Porcine retinal endothelial cells (PRECs) were isolated and cultured as described previously [28]. Briefly, freshly isolated retinas from porcine eye were washed and cut into 3 mm segments and transferred to a tube containing 4 ml of an enzyme cocktail (1 ml/retina) which consisted of 500 μg/ml collagenase type-IV (Sigma), 200 μg/ml DNase (Sigma) and 200 μg/ml pronase (Sigma) in 10 mM phosphate buffered saline containing 0.5% bovine serum albumin (BSA) at 37°C for 30 min. The resultant enzyme digests were passed through 53 μm steel mesh (W.S Tyler, UK). The trapped blood vessels were washed three times with minimal essential medium (MEM: Sigma St Louis, MO) by centrifugation at 400 × g for 5 min. The pellet containing microvessel fragments were finally suspended in Iscove's Modified Dulbecco's Medium (IMDM: Sigma St Louis, MO) with growth supplements on 35 × 10 mm culture dish coated with 1.5% gelatin type-A and incubated at 37°C with 5% CO_2_.

### Cell viability assay

The 3-(4, 5-dimethylthiazol-2-yl)-2, 5-diphenyltetrazolium bromide dye reduction assay using 96-well microtiter plates was performed according to the manufacturer's instructions (Roche Diagnostics, Mannheim, Germany). The assay relies on the reduction of MTT by the mitochondrial dehydrogenases of viable cells to yield a blue formazan product, which can be measured using a scanning multiwell spectrophotometer (Biorad, Model 680, Japan). PRECs were seeded at a density of 2 × 10^3 ^cells per well into 96-well culture plates and starved in IMDM with 0.5% serum for 5 h. To examine the effect of Ag-NP, VEGF-165B (Abcam, Cambridge, UK) and IL-1β (Abcam, Cambridge, UK) on cell viability, PRECs were treated with various concentrations of Ag-NP (from 0.1-1000 nM), VEGF and IL-1β and incubated for 24 h. After 24 h of incubation (37°C, 5% CO_2 _in a humid atmosphere), 10 μl of MTT (5 mg/ml in PBS) was added to each well, and the plate was incubated for a further 4 h (at 37°C). The produced formazan was dissolved in 100 μl of the dissolving buffer (provided as part of the kit) and absorbance of the solution was read at 595 nm. All measurements were carried out in triplicate.

### Pharmacological inhibitor assay

To assess the Src activity, the pharmacological inhibitor PP2 (Calbiochem, Germany) was used. Briefly, PRECs were seeded at a density of 2 × 10^3 ^cells per well into 96-well culture plates and starved in IMDM with 0.5% serum for 5 h. Cells were incubated with 10 μM of PP2 for 30 min before treatment with VEGF-and IL-1β. The assays were conducted over a 24 h incubation period at 37°C in a 5% CO_2 _incubator, and cell permeability was assessed.

### Plasmid constructs and transient transfection assay

The mutants at Lys295 (Kinase-deficient HA-Src KD K295 M) and Tyr527 (constitutive-active HA-Src-CA Y527F) were kindly provided by our collaborators and the constructs were employed as reported earlier [29]. PRECs were transiently transfected using nucleofection technique (Amaxa Biosystems, Koeln, Germany) and cultured to 80% confluence in IMDM medium. Briefly, cells were harvested by trypsinization and centrifuged at 1,500 × g for 10 min. The pellet was resuspended in the nucleofector solution (Basic nucleofector kit, Amaxa Inc, Germany) to a final concentration of 4-5 × 10^5 ^cells/100 μl. At the time of transfection, 1-3 μg of DNA encoding green fluorescent protein (pmaxGFP), constitutively active Src or dominant negative Src was added along with nucleofector solution and then subjected to electroporation using a nucleofector device-II (Amaxa Biosystems, Koeln, Germany: Program M-003) according to manufacturer's instructions. After electroporation, transfected cells were resuspended in 35 × 15 mm gelatin coated dishes containing 1 ml of prewarmed IMDM media and incubated in 5% CO_2 _at 37°C. The transfection efficiency was about 80-90% determined using pmaxGFP plasmid (Amaxa Biosystems) and cell viability determined by trypan blue exclusion was about 90%.

### Transwell monolayer permeability assay

To measure solute flux across endothelial cells, retinal endothelial cells were seeded onto 12-mm diameter Transwell filter inserts with a 0.4 μm pore size (Corning Inc); the inserts were placed into 12-well tissue culture plates. In some experiments, cells were first transfected with mutant Src constructs and then transferred to chambers. Chambers were examined microscopically for confluence, integrity, and uniformity of endothelial cell monolayers. 10 μM of rhodamine isothiocyanate (RITC)-dextran (70-kDa) (Sigma St Louis, MO) were applied to the apical chamber of the transwell inserts with a confluent endothelial cell monolayer. Growth factors were added for the designated times. Where applicable, Ag-NP was added 30 minutes prior to VEGF and IL-1β treatments. In some experiments, Src inhibitors were added to endothelial cell cultures 30 min prior to growth factor addition. The media volumes used equalized fluid heights in the apical and basolateral chambers, so that only diffusive forces were involved in solute permeability. At the indicated times after cytokine treatment, 100 μl samples were taken from the basolateral chamber and placed in a 96-well plate. A sample was taken from the apical chamber at the last time point; the amount of fluorescence in this chamber did not change significantly over the course of the experiment. Aliquots were quantified using a fluorescence multiwell plate reader (Biotek, Vermount, USA)

### Quantification of phospho-Src Y419 in cell lysate

Concentrations of phospho-Src were quantified by using a human phosphor-Src (Y419) ELISA kit based upon peptide competitive analysis(R & D systems, Minneapolis, MN) as per manufacturer's instructions. Briefly, 1 × 10^7^cells were seeded in a 60 mm tissue-culture dish and grown for 24 h. After the cells had attached and grown to confluence, the monolayer was starved for 6 h in IMDM with 0.5% FBS. After various treatments, cells were washed with 1× PBS (centrifuged at 2,000 × g, 10 min) and lysed using lysis buffer containing 1 mM EDTA, 0.05% Triton X-100, 5 mM NaF, 6 M Urea, 5 mM PMSF, 1 mM Na_3_VO_4_, 2.5 mM sodium pyrophosphate and a protease inhibitors (Sigma St. Louis, MO). After centrifugation at 2,000 × g for 10 min at 4°C, the supernatant containing proteins was removed and 6- fold dilution was made with buffer containing 1 mM EDTA, 0.5% Triton X-100, 1 M urea in 1× PBS. 100 μl of samples was added to each well of 96-well microplate coated with phospho-Src (Y419) capture antibody and incubated for 2 h at room temperature. After incubation, the plate were washed twice with PBS and incubated in blocking solution for 30 min. Following another wash with PBS, cells were incubated with the phospho-Src (Y419) detection antibody for 2 h at room temperature. After washing, 100 μl of streptavidin-HRP was added into each well and incubated for 20 min and then, 100 μl tetramethylbenzidine/H_2_O_2 _was added to the plates followed by the addition of 50 μl of stop solution. Colour formation was measured at an absorbance of 450 nm using a plate reader, which is directly proportional to the concentration of phospho-Src in the samples. The concentration of phospho-Src was determined using a calibration curve by generating a four parameter logistic curve fit.

### Transmission electron microscopy (TEM) analysis

TEM sample preparation involving cells, however, was performed by treating cells with silver nanoparticles for 6 h with under serum-free conditions. After the incubation, PRECs were centrifuged initially at 2,500 × g for 10 min. The resultant cell pellets were then washed thrice with PBS, and fixed in Trump's fixative (1% glutaraldehyde and 4% formaldehyde in 0.1 M phosphate buffer, pH 7.2). Thin section (90 nm) of samples for transmission electron microscopy (TEM) analysis were prepared on carbon-coated copper TEM grids and stained with lead citrate. TEM measurements were performed on a JEOL model 1200EX instrument operated at an accelerating voltage of 120 kV.

### Statistical analysis

All results were expressed as the mean ± standard error of the mean (SEM) values. Statistical significance difference was evaluated using ANOVA followed by paired two-tailed Student's t-test to compare with control group. A significance level of P < 0.05 was considered to be statistically significant.

## Results

### Characterization of silver NPs

**Figure 1:**
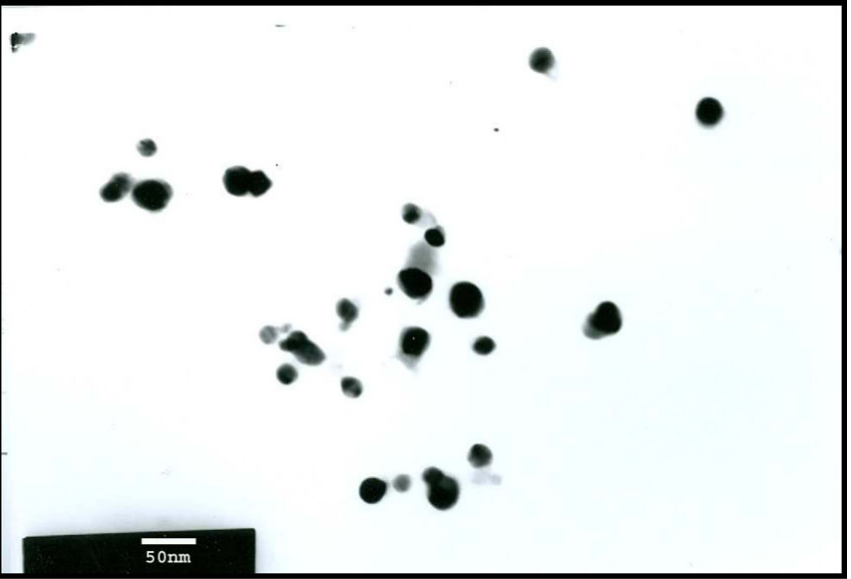
**TEM images obtained from purified fractions collected after sucrose density gradient of Ag-NPs synthesized using *B. licheniformis***. Purified nanoparticles from *B. licheniformis *were examined by electron microscopy. Several fields were photographed and were used to determine the diameter of nanoparticles. The range of observed diameters is 50 nm.

Prior to the study of anti-permeability effect of silver NPs, characterization of synthesized silver NPs was performed according to the methods described previously [23,24]. Silver NPs were synthesized using *B. licheniformis*. The synthesized nanoparticles were primarily characterized by UV-Visible spectroscopy, which has proved to be a very useful technique for the analysis of nanoparticles [24]. In UV-Visible spectrum a strong, broad peak, located at about between 440 nm was observed for silver NPs prepared using the biological system. Observation of this peak, assigned to a surface plasmon, is well documented for various metal nanoparticles with sizes ranging from 2-100 nm. Further characterization was carried out using particle analyzer. The results show that the particles range in size from 40 to 50 nm [24]. Transmission electron microscopic images show that purified nanoparticles are spherical with a mean diameter of 50 nm (Fig. 1).

### Cytotoxic effects of silver nanoparticle on PRECs

**Figure 2:**
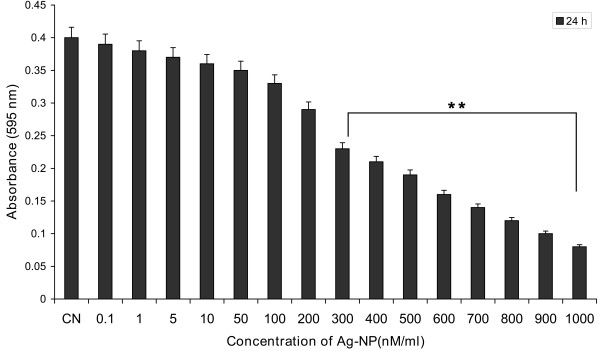
**Dose dependent effect of Ag-NP on PRECs viability**. PRECs were seeded in 96-well plate at a density of 2 × 10^3 ^cells/well and grown to confluence. After reaching confluence, cells were treated with the indicated Ag-NP doses, and the cell viability was measured by MTT assay after 24 h. Values are expressed in mean ± SEM, with each condition performed at least in triplicate (n = 3, **P < 0.05 Vs control).

To determine the cytotoxicity of Ag-NP, PRECs were exposed to various concentrations of Ag-NP for 24 h. Cell viability was measured by MTT assay as described in materials and methods. The results showed a dose-response increase in the cytotoxicity of Ag-NP on endothelial cells; exposure of cells to above 500 nM of Ag-NP caused significant cell death (Fig. 2). These results demonstrate that Ag-NP mediate dose dependent increase in toxicity. Since low concentrations of Ag-NP were found to be non-toxic, further studies of the effect of Ag-NP on vascular permeability were carried out using 100 nM of Ag-NP.

### VEGF and IL-1β increase permeability of retinal endothelial cells in a dose-dependent manner

**Figure 3:**
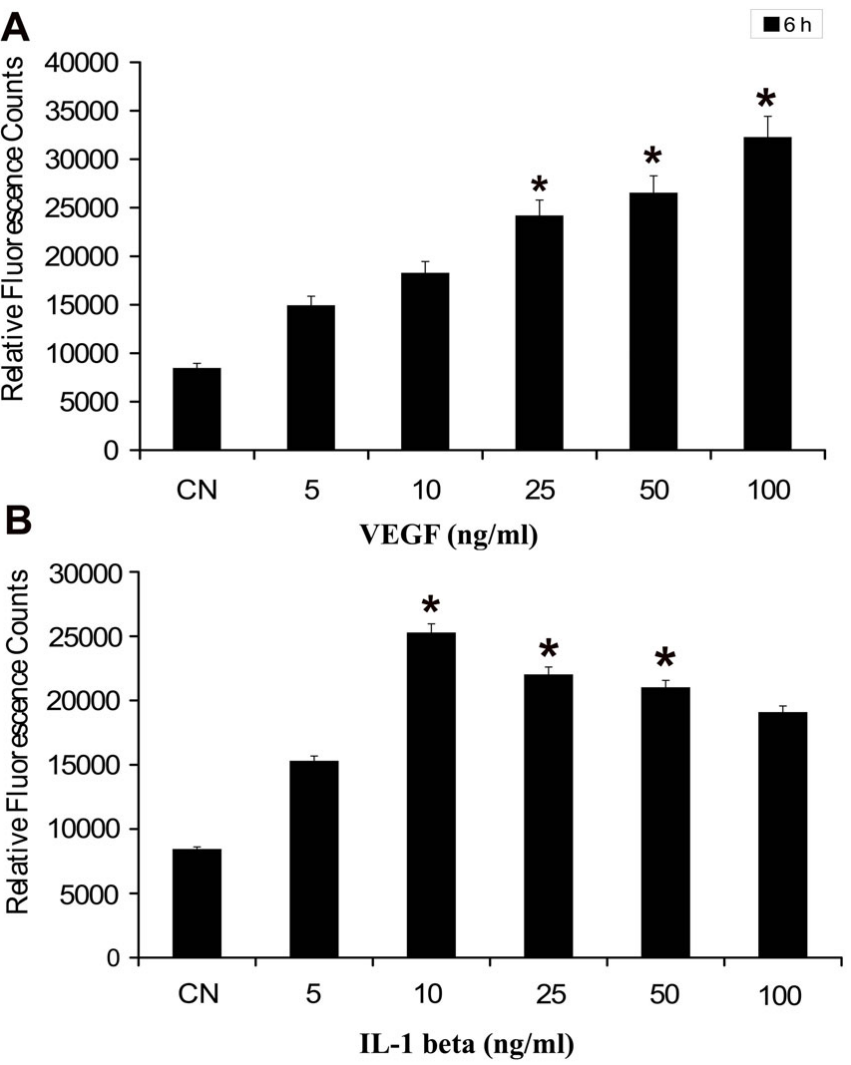
**Effect of VEGF and IL-1β on endothelial cell permeability**. PRECs were grown to confluent monolayers on porous membranes (12-well transwell insert plate) and treated with various concentrations of VEGF (A) and IL-1β(B). The flux of RITC-dextran from the upper to the lower chamber was measured after 6 h of treatment. Both VEGF-and IL-1β-induced endothelial cell permeability in dose-dependent manner. Values are expressed in relative fluorescence counts (RFUs) as mean ± SEM, with each condition performed at least in triplicate (n = 3, *P < 0.05 vs control). The figure is representative of three experiments with similar results.

In order to evaluate VEGF-and IL-1β-induced vascular permeability, we first characterized the effects of VEGF and IL-1β on permeability of retinal endothelial cells in our experimental system. PRECs were grown to confluence on transwell filters. They were then treated with different concentrations of VEGF and IL-1β. Both proteins induced a dose-dependent increase in permeability of the endothelial cell monolayer to RITC-labelled dextran at 6 h with maximal permeability observed at 100 ng/ml of VEGF (Fig. 3A) and 10 ng/ml of IL-1β (Fig. 3B). The effect of VEGF and IL-1β on permeability persisted over the 24 h duration of the experiment (data not shown). To study the effect of Ag-NP on vascular permeability we further used low concentrations of VEGF or IL-1β at, similar to those found in normal healthy human vitreous (25 ng/ml of VEGF and 10 ng/ml of IL-1β).

### Silver nanoparticles inhibit VEGF-and IL-1β-induced cell viability in PRECs

**Figure 4:**
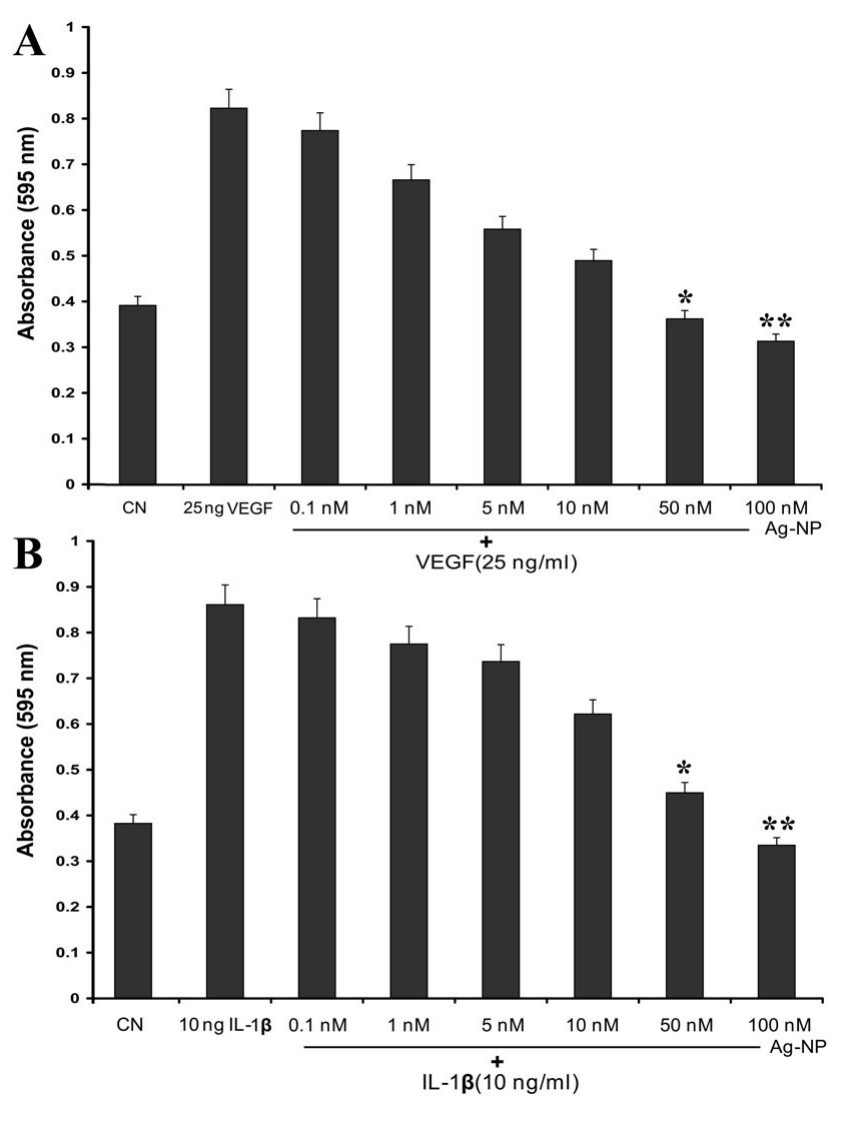
**Effect of various concentration of Ag-NP on VEGF and IL-1β-induced endothelial cell viability**. PRECs were seeded in 96-well plate at a density of 2 × 10^3 ^cells/well and grown to confluence. After reaching confluence, PRECs treated with indicated concentration of Ag-NP in presence of 25 ng/ml VEGF (A) and presence of 10 ng/ml IL-1β (B) for 24 h and the cell viability was measured by MTT assay. 100 nM Ag-NP significantly reduced the VEGF and IL-1β-induced cell proliferation (2 fold). Data are mean ± SEM representing similar results was obtained in three independent experiments (n = 3,*P < 0.05 vs VEGF and IL-1β treatment, **P < 0.01 vs VEGF and IL-1β treatment).

To measure the anti-angiogenic property of Ag-NP, the ability of Ag-NP to inhibit the VEGF-and IL-1β-induced endothelial cell proliferation was investigated by the MTT assay. Fig. 4A shows dose dependent effect of Ag-NP on VEGF-induced proliferation of PRECs. The addition of 100 nM Ag-NP with 25 ng/ml of VEGF significantly decreased cell proliferation (~60%, P < 0.01), if compared to the proliferation with VEGF alone. Similarly, the addition of Ag-NP (100 nM) with IL-1β (10 ng/ml) is also significantly decreased endothelial cell proliferation (P < 0.01) compared to the control level (Fig. 4B). A decline in cell survival was observed with lower concentrations of Ag-NP (0.1, 1,5,10 and 50 nM) treated with VEGF and IL-1β, but this was not significant compared with the effect of 100 nM Ag-NP. These data suggest that increasing concentrations of Ag-NP inhibits VEGF-and IL-1β-induced cell proliferation significantly.

### Silver nanoparticles blocks VEGF-and IL-1β-induced permeability

**Figure 5:**
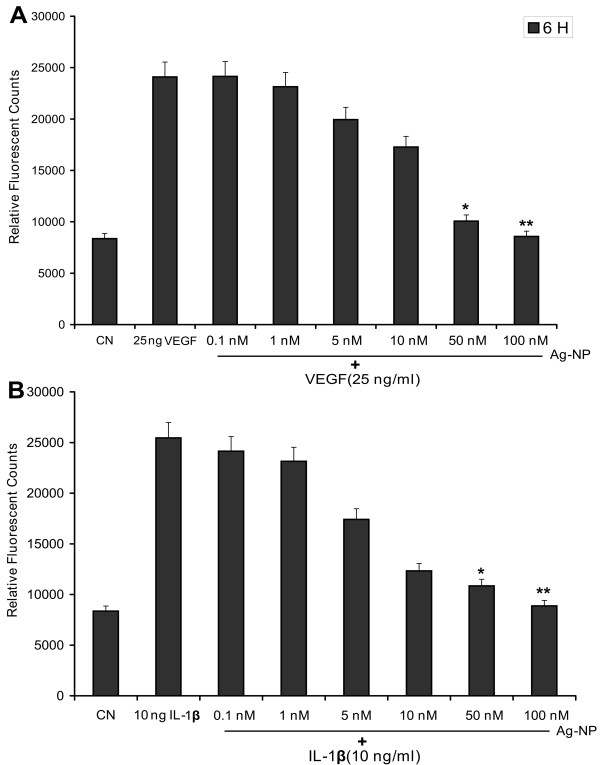
**Ag-NP inhibits the VEGF-and IL-1β-induced endothelial cell permeability**. PRECs were grown to confluent monolayers on porous membranes and were incubated with various concentration of Ag-NP with either 25 ng/ml VEGF(A) or 10 ng/ml IL-1β (B), and the flux of RITC-dextran from the upper to the lower chamber was measured 6 h after the treatment. Ag-NP were added 30 min prior to VEGF and IL-1β treatments. Pre-treatment with Ag-NP reduced the VEGF-and IL-1β-induced permeability to the level of control (0.5% serum) in a dose dependent fashion. Values are expressed in relative fluorescence counts (RFCs) as means ± SEM, with each condition performed at least in triplicate (n = 3,* P < 0.05 vs VEGF and IL-1β treatment, **P < 0.01 vs VEGF and IL-1β treatment).

Recent studies have demonstrated that nanogold blocks the activity of heparin-binding growth factors like VEGF165 and basic fibroblast growth factor (bFGF), whereas it does not inhibit the activity of non-heparin-binding growth factors like VEGF121 and endothelial growth factor [30]. To determine the role of Ag-NP on endothelial cell permeability, we next examined the possible inhibitory effect of various concentration of Ag-NP on the VEGF-and IL-1β-induced endothelial cell permeability. In this experiment, Ag-NP was added 30 min prior to growth factor treatment. Ag-NP inhibition of growth factor-induced permeability occurred in a dose-dependent fashion; 100 nM Ag-NP was sufficient to inhibit VEGF (Fig. 5A) and IL-1β (Fig 5B)-induced permeability significantly (P < 0.01) to the level of control. Doses lower than 100 nM Ag-NP did not block the VEGF-and IL-1β-induced permeability significantly. This result suggests that Ag-NP completely abrogated the VEGF-and IL-1β**-**induced increase in permeability.

### Src mediates VEGF- and IL-1β induction of endothelial cell permeability

**Figure 6:**
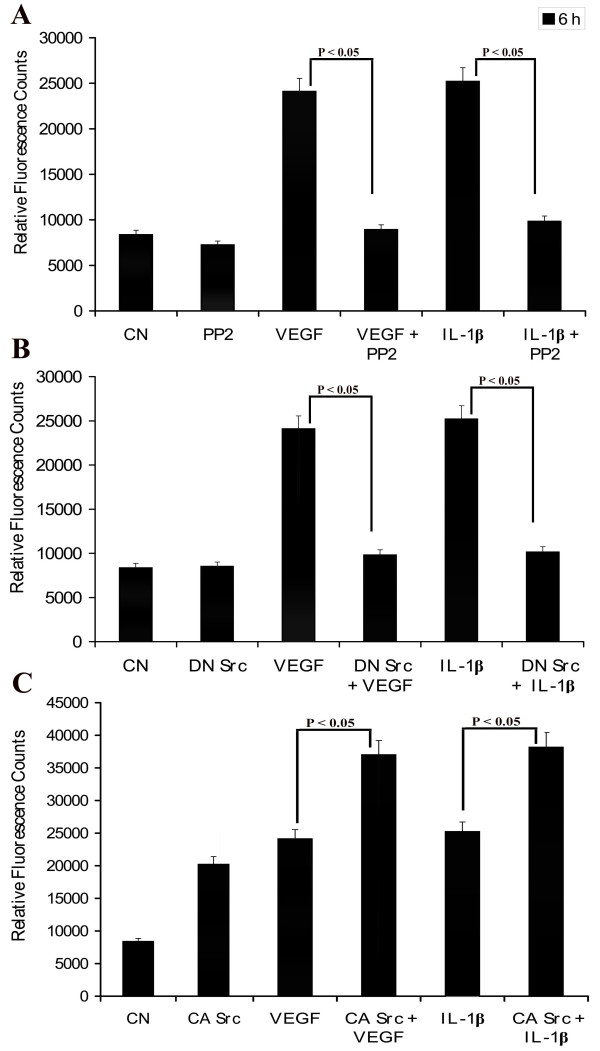
**Role of Src kinase activity in VEGF-and IL-1β-induced endothelial cell permeability**. (A) Effect of Src inhibitor on VEGF-and IL-1β-induced endothelial cell permeability. PRECs were grown to confluent monolayers on porous membranes (12-well transwell insert plate). The lower chamber was incubated with VEGF (25 ng/ml) or IL-1β (10 ng/ml) and in the presence or absence of PP2 (10 μM) for 6 h at 37°C; (B-C) PRECs were transiently transfected with DNA dominant negative Src (HA-Src KD K295 M) and constitutive active Src (HA-Src-CA Y527F). Transfected PRECs were treated with VEGF and IL-1β for 6 h at 37°C where the induction of permeability by growth factor in wild type cells was completely blocked in DN Src transfected cells (B) where the CA Src transfected cells resulted in increased permeability than the wild type cells(C). The flux of RITC-dextran from the upper to the lower chamber was measured 6 h after treatment. Values are expressed in relative fluorescence counts (RFCs) as mean ± SEM, with each condition performed at least in triplicate.

It has previously been demonstrated that Src family kinases (particularly Src and Yes) play a critical role in mediating VEGF-induced permeability *in vivo *[31]. Further, to depict the pathway involved in blocking the permeability of PRECs by Ag-NP, the potential involvement of Src kinase was investigated using PP2 inhibitor. PRECs were grown to confluence, and solute flux was determined in the presence of VEGF, IL-1β and PP2 (Src kinase inhibitor) at different combination. VEGF and IL-1β increased the cellular permeability whereas PP2 reduced the permeability. PRECs were pre-incubated with 10 μM PP2 inhibitor for 30 min, and then challenged with VEGF (25 ng/ml) and IL-1β (10 ng/ml) for 24 h. It was found that pre-treatment with PP2, inhibited endothelial cell permeability in the presence or absence of growth factor treatment (Fig. 6A). To further support the role of Src kinase activity in endothelial cell permeability, we performed transient transfection experiments of plasmid constructs expressing a dominant-negative mutant (kinase-deficient HA-Src KD K295 M) (DN Src) or constitutively-active Src (constitutive active HA-Src-CA Y527F) (CA Src). We first confirmed the effect of these mutants on permeability status in the presence or absence of VEGF and IL-1β. Analyses of serum-deprived PRECs revealed that transfection of CA Src increases endothelial cell permeability whereas transfection of DN Src decreased the cell permeability. Overexpression of DN Src blocked both VEGF-and IL-1β-mediated permeability to the level of control (Fig. 6B), whereas over expression of CA Src leads to a substantial increase in the endothelial cell permeability; stimulation of these cells with VEGF and IL-1β treatment had a synergistic effect on cell permeability which indicates that Src activation is sufficient for the induction of endothelial cell permeability (Fig. 6C). These data suggest that Src family kinase activity plays an important role in mediating the permeability effect of VEGF and IL-1β.

### Over-expression of constitutively active Src rescues Ag-NP- induced permeability

**Figure 7:**
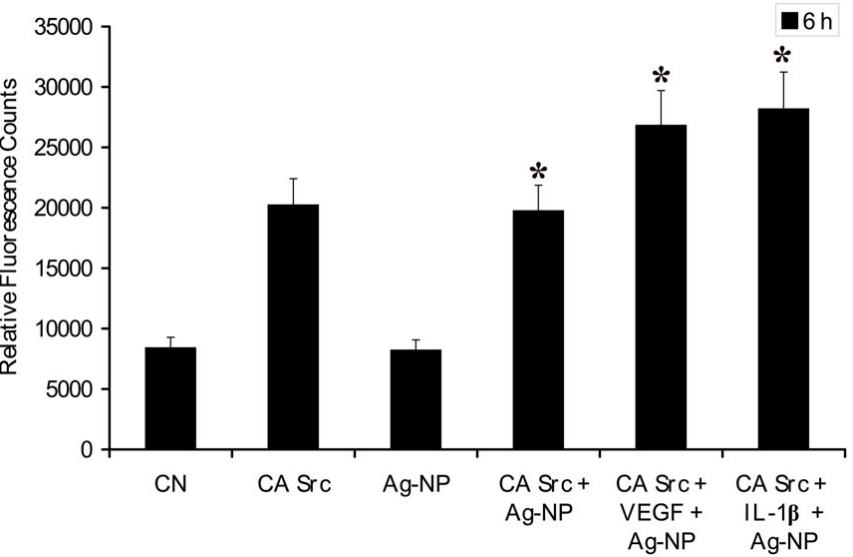
**Anti-permeability effect of Ag-NP is reversed by over-expression of constitutively active Src kinase**. PRECs were transiently transfected with dominant negative Src (HA-Src KD K295 M) and constitutive active Src (HA-Src-CA Y527F). Transfected PRECs were grown to confluent monolayers on porous membranes and then incubated with or without growth factors and Ag-NP for 6 h at 37°C, where the block in permeability by Ag-NP in wild type cells was overcome in CA-Src transfected cells. Ag-NP was added 30 min prior to growth factor. The flux of RITC-dextran from the upper to the lower chamber was measured 6 h after treatment. Values are expressed in relative fluorescence counts (RFCs) as mean ± SEM, with each condition performed at least in triplicate.

To determine whether modulation of Src was responsible for the observed effect of Ag-NP on cellular permeability, PRECs were transfected with a plasmid expressing a constitutively active form of Src. Transfected cells were serum starved and stimulated with VEGF and IL-1β in the presence or absence of Ag-NP and the dextran permeability assay was performed. Consistent with a role for Src kinase activity in endothelial cell permeability, PRECs transfected with CA Src displayed significant increase in permeability in the absence of VEGF and IL-1β treatment. Ag-NP was unable to block the permeability in cells transfected with CA Src, irrespective of cells being treated with or without VEGF and IL-1β (Fig. 7). Both the previous experiments utilizing dominant negative and constitutively active Src mutants, together with the results demonstrating Ag-NP inhibition of Src activation by VEGF and IL-1β, suggest that Ag-NP inhibit VEGF-and IL-1β-induced permeability effect on PRECs via blockade of the Src pathway.

### Ag-NP blocks the VEGF-and IL-1β-induced Src phosphorylation (Y419) in PRECs

**Figure 8:**
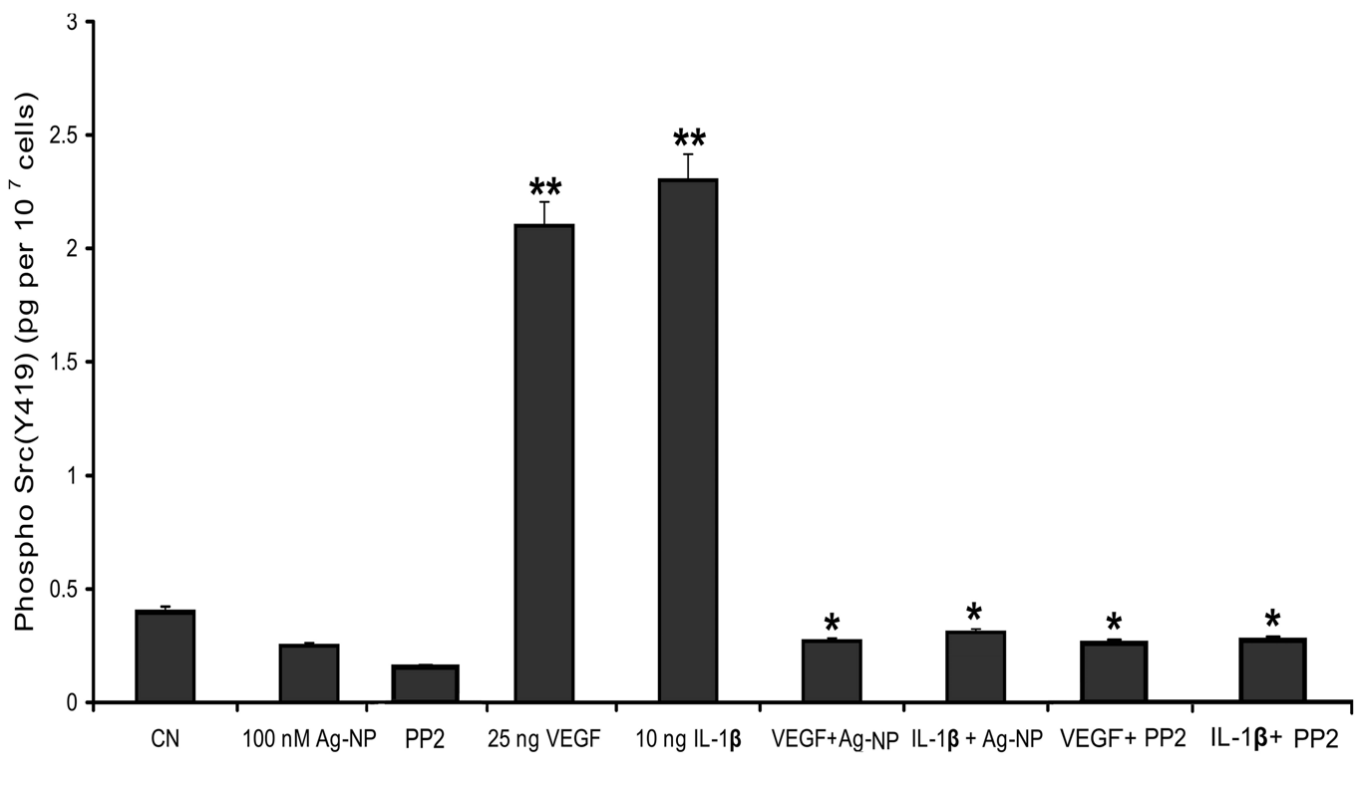
**Effect of Ag-NP and PP2 on VEGF-and IL-1β-induced Src phosphorylation**. PRECs were treated with VEGF and IL-1β in presence and absence of 100 nM Ag-NP or 10 μM PP2 for 1 hour. Level of Src phosphorylation (Y419) in cell lysate (1 × 10^7 ^cells) was checked by sandwich ELISA. VEGF (25 ng/ml) or IL-1β (10 ng/ml) treatments significantly increase the Src phosphorylation compared to the control. Both Ag-NP and PP2 significantly decreased the VEGF-and IL-1β-induced Src phosphorylation in PRECs. Data are means ± SEM representing similar results was obtained in three independent experiments (n = 3, *P < 0.05 vs control, **P < 0.01 vs control).

To support the contention that effects of Ag-NP and PP2 inhibitor on VEGF-and IL-1β-induced permeability were specifically directed through the Src pathway, we performed phospho-Src peptide competition immunoassay to measure the status of Src phosphorylation at Y419. Levels of phosphorylated Src (Y419) protein in the cell extracts were significantly increased after VEGF and IL-1β treatments compared with the control where as Ag-NP decreased Src phosphorylation in PRECs. The increased phospho-Src (Y419) form after VEGF (25 ng/ml) or IL-1β (10 ng/ml) treatment was significantly decreased by the pre-incubation of 100 nM Ag-NP (Fig. 8). In addition, significant changes of phosphorylated Src were observed in VEGF or IL-1β treated with PP2 (Src inhibitor). These data indicate that Ag-NP inhibit VEGF-and IL-1β-induced endothelial cell permeability through the inhibition of phospho-Src (Y419) activation.

### Src modulates the blocking effect Ag-NP on VEGF -and IL-1β-induced Src phosphorylation (Y419)

**Figure 9:**
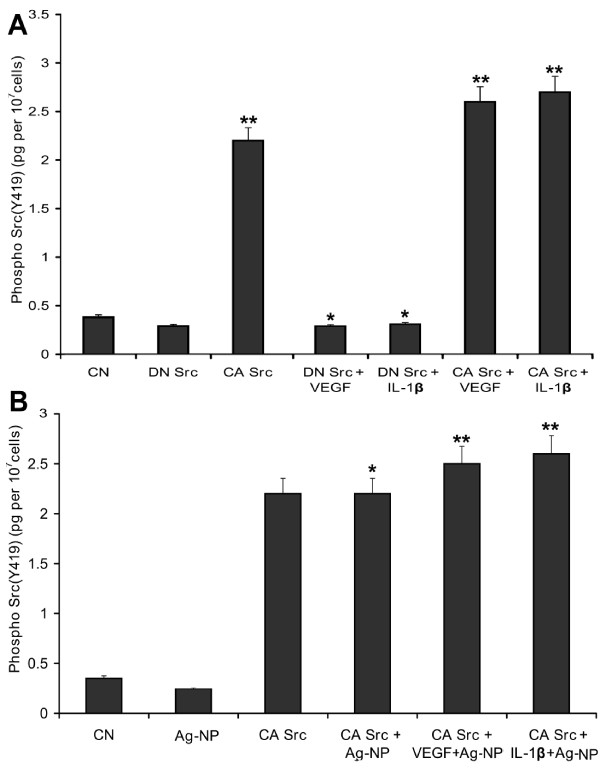
**Src modulates the inhibitory action of Ag-NP on VEGF-and IL-1β-induced Src phosphorylation (Y419)**. PRECs were transiently transfected with dominant negative Src (HA-Src KD K295 M) and constitutive active Src (HA-Src-CA Y527F). (A) Shows the effect of Src mutants on VEGF-and IL-1β-induced Src phosphorylation. DN Src mutant significantly blocks the VEGF-and IL-1β-induced Src phosphorylation whereas CA Src had an additive effect on Src phosphorylation. (B) Shows the Ag-NP rescues the inhibitory effect of Src phosphorylation in CA Src mutant. CA Src mutant confers resistance to Ag-NP blocking effect on VEGF-and IL-1β-induced Src phosphorylation at Y419. Data are means ± SEM representing similar results was obtained in three independent experiments (n = 3, *P < 0.05 vs control, **P < 0.01 vs control).

To confirm the central role of the Src pathway as a target for the anti-permeability effect of Ag-NPs, PRECs were transfected with a plasmid encoding DN Src and CA Src, followed by a treatment with VEGF and IL-1β in the presence or absence of Ag-NP and the level of Src phosphorylation were quantified by ELISA. Overexpression of DN Src reduced VEGF-and IL-1β-mediated Src phosphorylation at Y419 does to the level of control, whereas over expression of CA Src lead to a substantial increase in the Src phosphorylation. Stimulation of these cells with VEGF and IL-1β treatment had an additive effect on Src phosphorylation at Y419 (Fig. 9A). Overexpression of the CA Src completely counteracted the inhibitory effect of Ag-NP on VEGF-and IL-1β-induced Src phosphorylation (Y419) (Fig. 9B). We can conclude therefore that Ag-NP directly block VEGF-and IL-1β-induced Src phosphorylation on PRECs and controls cellular permeability through the inhibition of Src activation.

## Discussion

Vascular endothelial barrier dysfunction characterizes a diverse array of disease processes and plays an important pathophysiological role in many diseases including diabetic retinopathy [1]. The development of new therapeutic strategies aimed at reducing excessive vasopermeability could therefore have serious clinical implications. In particular, the characterization of new molecules with anti-permeability properties and elucidation of their mechanisms of action could facilitate efficient treatments.

Neovascularization occurs when there is an increase in the level of angiogenic factors like VEGF. Blood vessel formation is a complex phenomenon which involves a multi-step process that includes activation by angiogenic molecules, release of degradative enzyme production, migration and proliferation. To characterise potential anti-angiogenic activity of silver nanoparticles, their effects on the different steps involved in angiogenesis must be investigated. As endothelium is the target for many therapies, in the recent work we have demonstrated the effect of biologically-synthesized silver nanoparticles on VEGF-induced cell proliferation and migration in bovine retinal endothelial cells (BRECs) [32]. Silver nanoparticles of near-uniform size (40-50 nm), synthesized by the bacterium, *Bacillus licheniformis *were, found to block the proliferation and migration in BRECs [24] and to induce apoptosis [32]. In the current study we investigated the effect of silver nanoparticles on retinal endothelial cell permeability.

As induction of permeability is one of the major problems in angiogenic related diseases, many molecules are under consideration for therapy. Angiopoietin 1, for instance, has been found to have an impressive effect in blocking blood vessel leakage in animal models [33,34]. Pigment epithelium-derived factor (PEDF) has recently emerged as a molecule that can regulate vascular permeability. PEDF is known to have strong anti-angiogenic effects *in vivo *[35] and to regulate endothelial cell actions such as migration, proliferation, and survival *in vitro *[36-38]. *In vivo *studies have demonstrated that PEDF blocks VEGF-induced vascular permeability in the retina [39]. But, one of the disadvantages of all these molecules is the cost of the final product.

**Figure 10:**
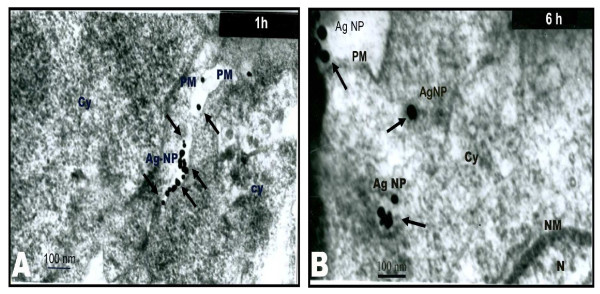
**TEM images of PRECs treated with silver nanoparticles**. (A) shows the image of the cells at 1^st ^hour and (B) shows the image of the cells taken at the 6^th ^hour. The latter image shows the silver nanoparticles internalized into PRECs. (where PM - plasma membrane, Ag-NP - silver nanoparticles, Cy - cytoplasm, NM - nuclear membrane, N - nucleus).

Recently gold nanoparticles have received great attention as an anti-angiogenic agent. It has been demonstrated that nanoparticles can block VEGF-induced retinal vascular permeability *in vivo *[30] Moreover, Elechiguerra et al. reported that silver nanoparticles with a size range of 1-10 nM bind with HIV-I virus [40]. We determined the effect of nanosilver in regulating endothelial cell permeability, using the solute flux assay. We found that Ag-NP were able to completely block retinal endothelial cell permeability induced by both VEGF and IL-1β; in addition, Ag-NP had a basal effect on reducing endothelial cell permeability. This suggests that Ag-NP may have a therapeutic role in the treatment of multiple conditions characterized by excessive permeability. The specific location of the nanoparticles during the treatment has been checked through various time intervals under a transmission electron microscope, which revealed that the nanoparticles of size ~50 nm were internalized during the treatment (Fig. 10). This correlates well with the previous reports where nanoparticles with size 50 nm were shown to be easily internalized compared to particles with other sizes [41]. Although the internalization of nanoparticles can be observed the subsequent effects are yet to be investigated. Here one possibility is blocking the activation of Src, which may block the VEGF-and IL-1β-induced permeability. With regard to signalling events mediating vascular permeability, the Src family kinases (particularly Src and Yes) have been demonstrated to play a critical role in mediating VEGF action [31]. Specifically, it has been demonstrated that VEGF-induced permeability in the dermal vasculature and brain was completely blocked in Src-deficient mice [31]. Previously, PP2 analogue was used to block the expression of IL-8 and VEGF, by blocking the Src kinase activation in tumour cells with high Src activity which was measured by the status of Src-phosphorylation at Y419 [42]. A similar finding was observed in Yes-deficient, but not Fyn-deficient mice [43]. Although IL-1β was shown to stimulate Src family kinase activity in other cell types (notably T-lymphocytes) [44], Src activation has not been reported for IL-1β in endothelial cells. Our results suggest that Src family kinase activity may be an important mediator for the well-documented vasopermeability effects of VEGF and IL-1β. Similar to the inhibitory effect of AP23846 on Src-kinase phosphorylation reported in [42], our study also demonstrated that treating PRECs with Ag-NPs blocked the Src-phosphorylation at Y419. Since Src-kinase activation plays a major role in inducing permeability, the ability of Ag-NP to block Src-kinase activation reduces cell permeability and indicates that Ag-NP may have a general regulatory effect on Src family kinase activity.

Our findings indicate that Ag-NP may have therapeutic benefits in addition to its anti-angiogenic properties [32]. The ability of Ag-NP to block both angiogenesis and permeability may render it uniquely beneficial as an agent of therapeutic choice for diverse complications. The production of VEGF by tumour cells may enhance metastasis by tumour cell extravasation from the bloodstream via endothelial barrier [45,46]. Disruption of Src signalling by pharmacologic blockade or by genetic approaches abrogated this VEGF effect. Therefore silver nanoparticles may have therapeutic potential in the treatment of cancer.

## Conclusion

Our findings indicate that Ag-NP may have potential therapeutic benefits in addition to their anti-angiogenic properties. Disruption of Src signalling by pharmacologic blockade or by Ag-NP approaches abrogated these VEGF and IL-1β effect. Our results indicate that Ag-NP have a therapeutic benefit in vascular permeability. Therefore Ag-NP may potentially provide attractive and cheap therapeutic alternative for treating various conditions characterized by excessive vasopermeability.

## Competing interests

The authors declare that they have no competing interests.

## Disclaimer

The opinions expressed in this article are those of the authors and do not necessarily represent any agency determination or policy.

## Authors' contributions

SS and KK performed the majority of the experiments. SG, SS and DV involved in writing the manuscript. SG, SE, and JP coordinated experiments, provided important advice for the experiments and financial support. SG, SS, KK and DV were involved with the design, interpretation and data analysis. All authors read and approved the final manuscript.
